# Maryland Bans Arsenical Drug in Chicken Feed

**DOI:** 10.1289/ehp.120-a269

**Published:** 2012-07-02

**Authors:** Charles W. Schmidt

**Affiliations:** Charles W. Schmidt, MS, an award-winning science writer from Portland, ME, has written for *Discover Magazine*, *Science*, and *Nature Medicine*.

Maryland has become the first state to ban arsenical feed additives used in chicken production.^1^ Signed 22 May 2012 by state governor Martin O’Malley, the ban applies mainly to a drug called roxarsone (sold as 3-Nitro^®^) and takes effect 1 January 2013.

Produced by Pfizer subsidiary Alpharma LLC and used for nearly 60 years to prevent and treat infections caused by parasites called coccidians, roxarsone also helps chickens grow faster with less feed. It’s not known how roxarsone kills coccidians, according to John Barta, a professor of parasitology at the University of Guelph, Canada.

At the time roxarsone was approved, scientists believed its organic arsenic base would be excreted unchanged. Then emerging research showed that birds can metabolize organic arsenic to the more toxic inorganic form.^2^ Furthermore, whereas fresh poultry waste does contain predominately organic arsenic, in the soil it degrades to inorganic arsenic, which has been found to run off into receiving waters and accumulate in stream sediments.^3^ In studies of these environmental media in the Delmarva Peninsula, spreading the waste of roxarsone-fed poultry on soil was associated with inorganic arsenic concentrations that occasionally exceeded federal and state re-med-iation standards; how-ever, deep ground-water, the major source of drinking water on the peninsula, appears to be largely unaffected by the application of manure containing arsenic.^3^ Pfizer spokesman Christopher Loder says, “We are not aware of evidence that demonstrates a risk to the environment from use of roxarsone.”

Maryland imposed the ban in 2012, the third consecutive year that such legislation was considered in the General Assembly. The latest effort followed a 2011 study by the U.S. Food and Drug Administration (FDA) that detected higher levels of inorganic arsenic in the livers of roxarsone-treated broiler chickens than in untreated controls.^2^ The FDA investigators assessed combined inorganic and organic arsenic in the meat and organs of treated and untreated birds. However, they completed a speciation profile—teasing apart inorganic and organic content—only for liver tissue.

**Figure:**
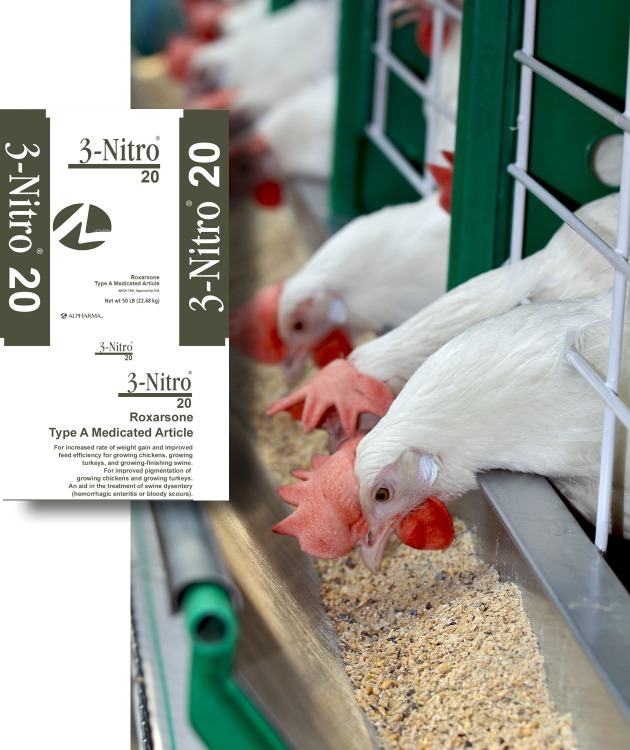
Coccidiosis is prevented in poultry by administering mixtures of anticoccidial drugs via feed. Some chicken producers have reported that removing roxarsone from the mix reduced the efficacy of certain anticoccidials that had historically been administered in combination with it. Some nonarsenical drugs may need to be used at the higher end of their approved ranges to achieve the same efficacy, says Bill Satterfield of Delmarva Poultry Industry, Inc. © Shutterstock.com; inset: dailymed.nlm.nih.gov

3-Nitro contains both the active ingredient roxarsone and a carrier that contains rice hulls, which themselves can be high in inorganic arsenic. The FDA analysis indicated that “it is unlikely that the rice hulls are contributing any significant amount of arsenic to the finished medicated diet.”^2^ However, Bill Satterfield, executive director of Delmarva Poultry Industry, Inc., a trade group based near Georgetown, Delaware, points out that the source of the inorganic arsenic was not determined in the FDA study nor was any transformation pathway demonstrated.

In July 2011 Alpharma voluntarily suspended 3-Nitro sales in the United States.^4^ The new ban therefore applies to roxarsone that poultry farmers had stockpiled for future use. But according to Satterfield, most Maryland chicken companies stopped using the drug after Alpharma stopped selling it.^5^ “Producers were given an opportunity to purchase limited amounts of roxarsone to help with the transition to a new product,” he says, “but from what I understand, no one is using it now.”

The Maryland ban exempts Histostat® (nitarsone), another Alpharma arsenical for use in poultry, which is the only FDA-approved treatment for histomoniasis, a potentially lethal illness. Moreover, according to Satterfield, the roxarsone ban can be lifted in Maryland in the event the FDA supports such an action in the future. FDA spokeswoman Laura Alvey says, “The agency continues to work with [Alpharma] to fully investigate the issue and, as part of this effort, FDA is conducting some additional confirmatory testing to address some remaining scientific questions. The suspension of sales will remain in effect as this work is completed.”

Inorganic arsenic, a known human carcinogen, has also been implicated in gastrointestinal, dermatologic, neurologic, reproductive, and cardiovascular effects in humans.^6^ However, experts acknowledge that the roxarsone withdrawal is not based on demonstrated cases of human illness resulting from eating treated chicken. Instead, it is driven chiefly by the Delaney Clause, a 1958 amendment to the Food, Drug, and Cosmetic Act^7^ stating that, with some exceptions, the FDA cannot approve known human carcinogens for use in the food supply. Perdue Farms,^3^ McDonald’s restaurants,^3^ and the Chipotle Mexican Grill restaurant chain^8^ are among companies that reportedly no longer grow or buy chickens given arsenical feed additives, which Barta says is further evidence of a growing trend away from prophylactic medications and antimicrobial growth promoters in the poultry industry.
